# Asymmetric Oxo‐Bridged ZnPb Bimetallic Electrocatalysis Boosting CO_2_‐to‐HCOOH Reduction

**DOI:** 10.1002/advs.202104138

**Published:** 2021-11-10

**Authors:** Aya Gomaa Abdelkader Mohamed, Enbo Zhou, Zipeng Zeng, Jiafang Xie, Dunfeng Gao, Yaobing Wang

**Affiliations:** ^1^ CAS Key Laboratory of Design and Assembly of Functional Nanostructures and Fujian Provincial Key Laboratory of Nanomaterials State Key Laboratory of Structural Chemistry Key Laboratory of Optoelectronic Materials Chemistry and Physics Fujian Institute of Research on the Structure of Matter Chinese Academy of Sciences Fuzhou Fujian 350002 China; ^2^ University of Chinese Academy of Sciences Beijing 100049 China; ^3^ Key Laboratory of Urban Pollutant Conversion Institute of Urban Environment Chinese Academy of Sciences Xiamen 361021 China; ^4^ Dalian National Laboratory for Clean Energy Dalian 116023 China; ^5^ Fujian Science and Technology Innovation Laboratory for Optoelectronic Information of China Fuzhou 350108 China

**Keywords:** bimetallic electrocatalyst, CO_2_ reduction, electrocatalysis, formate, selectivity

## Abstract

Electrochemical CO_2_ reduction (ECR) is one of the promising CO_2_ recycling technologies sustaining the natural carbon cycle and offering more sustainable higher‐energy chemicals. Zn‐ and Pb‐based catalysts have improved formate selectivity, but they suffer from relatively low current activities considering the competitive CO selectivity on Zn. Here, lead‐doped zinc (Zn(Pb)) electrocatalyst is optimized to efficiently reduce CO_2_ to formate, while CO evolution selectivity is largely controlled. Selective formate is detected with Faradaic efficiency (FE_HCOOH_) of ≈95% at an outstanding partial current density of 47 mA cm^–2^ in a conventional H‐Cell. Zn(Pb) is further investigated in an electrolyte‐fed device achieving a superior conversion rate of ≈100 mA cm^–2^ representing a step closer to practical electrocatalysis. The in situ analysis demonstrates that the Pb incorporation plays a crucial role in CO suppression stem from the generation of the Pb–O–C–O–Zn structure rather than the CO‐boosted Pb–O–C–Zn. Density functional theory (DFT) calculations reveal that the alloying effect tunes the adsorption energetics and consequently modifies the electronic structure of the system for an optimized asymmetric oxo‐bridged intermediate. The alloying effect between Zn and Pb controls CO selectivity and achieves a superior activity for a selective CO_2_‐to‐formate reduction.

## Graphical abstract

This study reports a novel Zn(Pb) electrochemical CO_2_ reduction (ECR) electrocatalyst that shows >95% selectivity to formate production at an outstanding partial current density of 47 and ≈100 mA cm^−2^ in H‐Cell and flow cell, respectively, representing practical electrocatalysis. In situ and theoretical studies explain the local chemical environmental evolution and reveal the alloying effect of Zn(Pb) for ECR‐to‐HCOOH conversion.

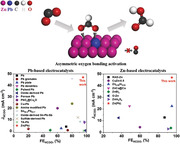

## 1 Introduction

Recent decades have witnessed the disturbance of the global carbon‐cycle balance and is likely to be ongoing. To meet this disturbance, avoiding fossil fuel production, for example, cannot be practicable because doing so negatively impacts both the world's industry and the technology revolution. Alternative approaches for sustaining the carbon balance and minimizing fossil fuel utilization include solar,^[^ ^1^ ^]^ hydrogen,^[^ ^2^, ^3^, ^4^ ^]^ carbon dioxide^[^ ^5^ ^]^‐based fuels production. Electrochemical CO_2_ reduction (ECR) is one of the promising CO_2_ recycling technologies with additional benefits toward green fuel generation. Among the expected ECR products, formate (a liquid fuel or fuel additive; HCOOH/HCOO^‐^) is highly desirable because it has the highest energy values (current market price per energy unit)^[^ ^6^ ^]^ having advantages in terms of handling, transportation, and storage, in addition, being non‐flammable and nontoxic supply for the electric energy generation. Metal‐based ECR electrocatalysts including In,^[^ ^7^, ^8^, ^9^, ^10^ ^]^ Sn,^[^ ^11^, ^12^, ^13^, ^14^ ^]^ Bi,^[^ ^15^, ^16^, ^17^ ^]^ and Pb^[^ ^13^, ^18^, ^19^ ^]^ are distinctive in their ability to selectively reduce CO_2_ into formate reaching Faradic efficiency (FE_HCOOH_) of >90%.^[^ ^20^ ^]^ It is worth noting that for Pb‐based electrocatalysts, the CO_2_‐to‐HCOOH activity performance still suffers from the relatively low partial current densities;*j*_formate_/j_HCOO_^‐^. Thus, sustaining formate generation during the ECR process with high activity is highly favorable for practical electrocatalysis.

Zn sites are attractive, nonprecious, nontoxic electrocatalysts with advantages toward inhibiting the competitive hydrogen evolution reaction (HER). However, Zn is a well‐known CO‐selective ECR electrocatalyst. The high CO selectivity on Zn sites is attributed to adsorb CO_2_ molecule via C‐bonding mode and further stabilize *COOH intermediate leading to CO release. The ECR selectivity toward another desired product (formate in our case) can be tuned by breaking the scaling relationships of commonly recorded metals.^[^ ^21^, ^22^, ^23^ ^]^ Formate‐selective Zn‐based electrocatalysts have been rarely reported, notably when applying more negative cathodic potentials, and also suffer from low CO_2_‐to‐HCOOH activities.^[^ ^24^, ^25^ ^]^ The key parameter to selectively reduce CO_2_ into formate on Zn sites is modifying the stability of *COOH intermediate and tune the chemical environment to adsorb CO_2_ via oxygen bonding and form the desired oxygen bridge intermediate (*OOCH) that can control the selectivity and enhance the reaction kinetics for formate production. Thus, formate selectivity is attributed to the oxophilicity characteristics with high oxygen bonding affinity of CO_2_ molecule, on the contrary with CO, boosting formate generation.^[^ ^22^, ^26^ ^]^

The bimetallic alloying strategy is interesting due to its potential ability to modulate the binding energy of intermediates on the active sites promoting certain chemical bonding and activating some atoms.^[^ ^26^, ^27^, ^28^, ^29^ ^]^ For instance, Pd‐Sn electrocatalyst was developed for selective ECR electrocatalysis toward formate versus CO generation.^[^ ^30^ ^]^ This performance was attributed to tuning the electronic state by Sn alloying and forming PdSnO_2_ oxide species on the surface of the PdSn/C catalyst which are favorable for the HCOO* intermediate‐involved pathway. Similarly, In‐Zn bimetallic electrocatalyst exhibited a considerable production rate of selective formate while suppressed CO production due to the suitable binding energy of *OCHO intermediate on the interfacial In‐Zn sites.^[^ ^25^ ^]^ These studies suggested that the electrocatalytic activity and selectivity highly depend on the catalyst composition and its relationship with the ECR intermediates regarding the mechanistic pathway.

Inspiring by cooperative active sites designs that could bring a favorable environment configuration effectually fasting the reaction kinetics, we became interested in modifying the Zn surface with a suitable reactive site in which the *COOH intermediate can be tuned into *OOCH configuration targeting maximizing the ECR activities. In this work, from the proof‐of‐concept perspective, a novel Pb doped Zn catalyst (Zn(Pb)) was developed by a facile co‐electrodeposition method as an excellent selective CO_2_‐to‐HCOOH electrocatalyst with an outstanding formate production activity under ambient conditions compared to Zn‐ and Pb‐based electrocatalysts. The interfacial structure was correlated with the intermediate stabilization based on the potential‐dependent in situ attenuated total reflectance‐Fourier transform infrared (ATR‐FTIR) detection and density functional theory/density of states (DFT/DOS) computations. Tuning the ECR intermediates from C‐bonded into asymmetric O‐bonded mode of CO_2_ adsorption on Zn(Pb) sites steer selective control of CO and an energetically favored environment for HCOO^–^ generation.

## 2 Results and Discussion

### 2.1 Zn(Pb) Synthesis and Characterization

**Figure 1:**
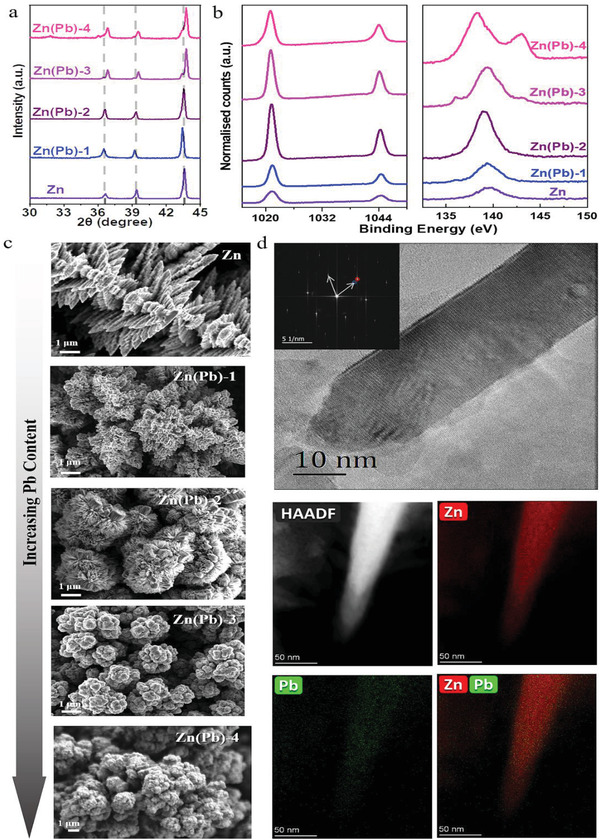
Structural analysis of Zn(Pb) series. XRD patterns a), XPS b), and SEM images c) of the undoped and doped Zn. d) HRTEM imaging of Zn(Pb)‐4 and HAADF image with the corresponding elemental mapping.

Zn(Pb) electrodes were prepared by a one‐step co‐electrodeposition strategy by which a series of Zn(Pb) films with controllable lead concentrations can be produced (Table S1, Supporting Information). X‐ray diffraction (XRD) measurements confirmed the Zn(Pb) structure to include metallic Zn, although the (002), (100), and (101) peaks are shifted toward higher angles and split with increasing Pb content,^[^ ^31^ ^]^ as shown in **Figure** **1**a. This trend refers to the compression and tension^[^ ^32^ ^]^ of Zn phases due to the embedded Pb atoms supported by the XRD simulation presenting a similar pattern, as shown in Figure S1 in the Supporting Information. The presence of lead in Zn(Pb) films is unambiguously confirmed using X‐ray photoelectron spectroscopy (XPS) (Figure 1b; Figure S2, Supporting Information). The presence of two metallic atoms was recorded according to XPS of Zn 2p and Pb 4f ( Figure S2, Supporting Information). The presence of lead in Zn(Pb) films was further confirmed using inductively coupled plasma optical emission spectroscopy (ICP‐OES) and EDS as listed in Table S1 in the Supporting Information. The Zn(Pb) films show a 3D porous dendritic morphology using the highly branched structure of Zn dendrites as a template,^[^ ^33^ ^]^ as shown in scanning electron microscope (SEM) images of Figure 1c. Such a 3D porous structure can facilitate reactants' mass transfer and provide abundant active sites for CO_2_ reactions. It should be noted that the Zn‐free Pb film showed a relatively different morphology structure in which non‐porous Pb particles with smooth and compact shapes can be observed in Figure S3 in the Supporting Information. This can explain the observed aggregate size with larger dendrites formation due to the increase of Pb content. High‐resolution transmission electron microscope (HRTEM) imaging of Zn(Pb) shows dendrites with nanometer‐sized needle‐like tips that are essentially made of two single crystals belonging to PbO and metallic Zn, as seen in Figure 1d. This oxide phase could be attributed to a gaseous O_2_ reaction with freshly prepared films during the drying process.^[^ ^34^ ^]^ High‐angle annular dark field (HAADF) and the corresponding elemental mapping reveal that Pb and Zn are uniformly distributed over the needle‐like tip of the dendrites in Zn(Pb).

### 2.2 Electrocatalytic Performance of the Zn(Pb) Electrodes

Electrochemical studies were performed in a two‐compartment H‐cell using 0.1 m KHCO_3_ as an electrolyte. Analysis of the product distribution showed that the ECR selectivity on Zn(Pb) electrodes is changed to generate formate replacing CO on Pb‐free Zn electrode, as represented in Figure S4a in the Supporting Information. The required overpotential to reach the highest FE_HCOOH_ increased with increasing Pb content showing a maximum FE_HCOOH_ nearly of 98% and 95% at −1.1 and −1.2 *V*_RHE_ on Zn(Pb)‐3 and Zn(Pb)‐4, respectively. To our best knowledge, this novel material has not been reported for formate‐selective Zn‐based electrocatalysts as listed in Table S2 in the Supporting Information. What is more, the surface structure‐selectivity correlation can be observed at a certain applied potential. At −1.1 *V*_RHE_, FE_HCOOH_ significantly increases while CO Faradic effeciency (FE_CO_) decreases from Zn(Pb)‐1 to Zn(Pb)‐3 which could be correlated with morphological changes observed in SEM imaging. CO product was significant over Zn (FE_CO_ of 88%) at −1.2 *V*_RHE_. On the other hand, the analysis of potential‐dependent partial current densities on Zn(Pb) electrodes exhibits an outstanding rate achieved at ‐1.2 *V*_RHE,_ recording the highest formate partial current densities (*j*_formate_/*j*_HCOO_^‐^) of ‐47 mA cm^–2^, as shown in Figure S4b in the Supporting Information. Excitingly, this activity rate of the formate production is six times that of similar reported dendritic morphology of Pb^[^ ^34^ ^]^ (7.5 mA cm^–2^). This confirms the effect of the alloying parameter to provide a favorable ECR‐to‐formate environmental configuration, nevertheless the morphological factor.

**Figure 2:**
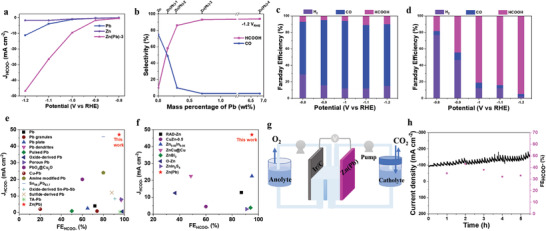
ECR performance: a) partial current densities of HCOO^–^. b) Selectivity as a function of Pb content. c,d) Corresponding FE for HCOO^–^, CO, H_2_ products on undoped and doped Zn, respectively. e,f) Comparison of the maximum FE and HCOO^–^ partial current density with reported Pb and Zn catalysts, respectively. g) Experimental setup of ECR flow‐cell experiment. h) The stability of ECR conversion activity toward HCOO^–^ generation and its corresponding FE.

To figure out the alloying advantage, the ECR performance will be discussed by comparing the Zn(Pb)‐3, Pb‐free Zn, and as prepared Zn‐free Pb electrodes. The stable current densities were plotted against potential, as represented in Figure S5a in the Supporting Information. As expected, the ECR kinetics have increased as the applied potential shifted in the negative direction. The potential‐dependent HCOOH partial current densities were calculated based on the total current densities and their corresponding FE_HCOOH_, as seen in **Figure** **2**a. It clearly reveals the high catalytic activity of Zn(Pb)‐3 toward formate formation, indicating faster kinetics compared to the substantial Zn‐free Pb electrode. To address the composition structure‐selectivity correlation, the dopant (Pb) content‐dependent selectivity was plotted, as shown in Figure 2b. Formate generation was selectively increased with increasing Pb content reaching a steady‐state starting 0.7% of Pb content. As shown in Figure 2c,d, the selectivity has changed from CO into HCOOH indicating the alloying outcome of the ECR performance on Zn sites. Consequently, the selective formate production could be attributed to the alloying effect of Zn/Pb surface acting as main active sites, while CO generation was suppressed due to well‐distributed Pb atoms affecting its binding energy on Zn sites. To be noted, HCOOH was the main product for smooth Pb electrode with FE_HCOOH_ of 34% lower than the Zn(Pb) (of 95%) at −1.2 *V*_RHE_ ( Figure S5b, Supporting Information). The electrochemical stability was measured, showing that Zn(Pb) maintains the FE_HCOOH_ at 90% against 7 h of time at −1.2 *V*_RHE_ , indicating a sustainable selectivity rate, as seen in Figure S6 in the Supporting Information. To our best of knowledge, such an increase in formate activities has not been reported on Pb‐ or Zn‐based electrocatalysts before, Tables S2 and S3 in the Supporting Information, as represented in Figure 2e,f. These findings verify the key role of the alloying effect in controlling the CO selectivity on Zn sites.

In a proof‐of‐principle feasibility of its practical use, we investigated the ECR performance of Zn(Pb) bimetallic cathode in a two‐electrode CO_2_‐bubbled electrolyte‐fed flow cell reactor as represented in Figure 2g; Figure S7 in the Supporting Information. The present flow cell has an advantageous configuration for getting rid of the issues related to a gas diffusion electrode (GDE) utilization and performing the ECR electrocatalysis in a continuous flow reactor avoiding the mass transfer limitations at ambient conditions. The ECR on Zn(Pb) in 0.5 m KHCO_3_ solution showed a FE_HCOOH_ of ≈30:35% with an excellent current density of ≈100 mA cm^–2^ stabilized along 5 h as shown in Figure 2h. The decrease in FE_HCOOH_ could be attributed to the specific design of the flow reactor in which the KHCO_3_ electrolyte is supplied as the main reactant with a low concentration of the dissolved CO_2_, HCO_3_^–^, and CO_3_^2–^ species, in addition, the local concentrations of H^+^ donors are still available and competitive around the electrocatalyst surface resulting in a reduction of the Faradic efficiency of formic acid.^[^ ^35^, ^36^ ^]^ This finding brings new prospects for realizing high CO_2_‐to‐HCOO^–^‐current densities on effective Zn(Pb) bimetallic electrocatalyst.

To uncover this performance, the double‐layer capacitance (*C*_dl_) was measured and presented in Figure S8a in the Supporting Information in which the slope value acts as a reference for the electrochemical surface area (ECSA). The density of electrochemically active sites of Zn(Pb) and Pb‐free Zn is relatively closer and about nine times that of Zn‐free Pb electrode. Large ECSA proves the benefits of the environmental configuration for the electrocatalytic activity.^[^ ^37^ ^]^ Thereby, it provides a piece of evidence that the Zn(Pb) and Pb‐free Zn have rich favorable electrochemical ECR environment to formate and CO, respectively, with more ECR active sites observed in product distribution measurements showing high catalytic performance regardless of the reaction product. Thus, the Zn(Pb) and Pb‐free Zn showed the best performance with the highest FE_HCOOH_ and FE_CO_, respectively, with exceptional current densities. To obtain kinetic insights, The Tafel plots were investigated and represented in Figure S8b in the Supporting Information. The Tafel slope, an indication of kinetics and involving rate‐determining step (RDS) for HCOOH formation, was 69.5 mV dec^–1^ for porous Zn(Pb) and non‐porous Zn‐free Pb at relatively high overpotential. Indeed, the measured HCOOH production rates at higher overpotential are strictly determined by electrokinetics, not nonkinetic factors, for example, mass transport limitations. Dunwell et al. have ascribed the proposed ECR reaction pathways and their corresponding Tafel slopes.^[^ ^38^ ^]^ Accordingly, Tafel measurements of as‐prepared samples are close to the theoretical Tafel value of 59 mV dec^–1^ for the RDS, indicating that the RDS is proton transfer step (Equation. 2) rather than the first electron transfer step (Equation 1), as follows

(1) $$*CO_{2}+\;e^{-}\;\to \;*{\mathrm{CO}}_{2}^{-}$$

(2) $$*{\mathrm{CO}}_{2}^{-}+\;H^{+}\to *\mathrm{COOH}/*\mathrm{OOCH}$$

Measuring the Tafel slope is beneficial for practical applications since it reflects a much faster rate to include ECR on their surfaces, as further confirmed by the findings of electrochemical impedance spectroscopy (EIS) measurements. The charge transfer resistance (*R*_ct_) for CO_2_ reduction was investigated and demonstrated in Figure S8c in the Supporting Information. It can be seen that the Nyquist plots of Zn(Pb) show a smaller semicircle than that of Pb‐free Zn and Zn‐free Pb electrocatalysts, indicating its lower polarization resistance toward ECR and further fast increase in charge transfer. Thus, the existence of Pb in the Zn(Pb) film decreases *R*_ct_ with respect to the Pb‐free Zn and Zn‐free Pb electrodes. The results could clearly promote the alloying‐dependent reaction kinetics, leading to remarkable ECR activity and selectivity.

### 2.3 Unveiling the Intermediates

**Figure 3:**
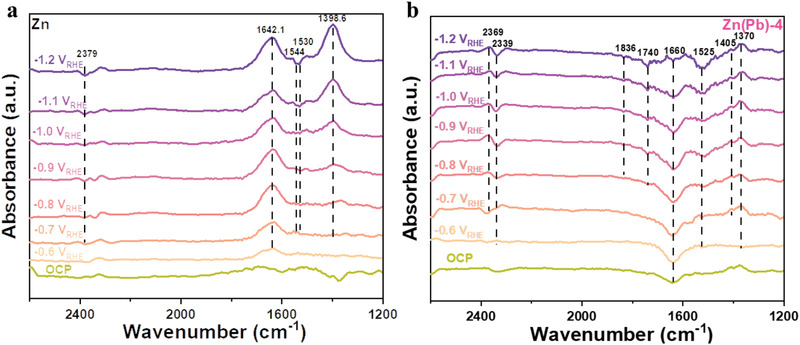
Potential‐dependent in situ ATR‐FTIR spectra of the undoped and doped Zn with CO_2_ purging.

The chemical structures of the intermediates were further identified by in situ ATR‐FTIR spectroscopy on Pb‐free Zn and Zn(Pb) electrocatalysts. As shown in **Figure** **3**, with applying potentials comparing to open circuit potential (OCP), the intensity of four bands of 2379/2369, 2339, 1398, and 1660 cm^–1^ could be observed, which can be assigned to the dissolved CO_2_ in the liquid layer,^[^ ^39^ ^]^ the adsorbed CO,^[^ ^39^ ^]^ the carbonate anions in the solution^[^ ^40^ ^]^ or the symmetrical stretch vibration of COO^–^ indicating to the adsorbed CO_2_,^[^ ^39^, ^41^, ^42^ ^]^ and the C═O stretch which can be fitted with the *OCOH (lay down *COOH) and *COOH intermediate frequency^[^ ^39^ ^]^ or OH deformation,^[^ ^43^ ^]^ respectively. Regarding the spectrum of Zn(Pb), shifting from a relatively weak 1544:1530 to near 1525 cm^–1^ bands can be found with increasing the applied potential, which could refer to the O‐C‐O species in the two‐oxygen bridge‐bonded formate intermediate (*OOCH)^[^ ^40^ ^]^ matching with the simulated FTIR, as listed in Table S4 in the Supporting Information. Two individual bands of 1370 and 1405 cm^–1^ which can be assigned to *COOH and a bidentate COO^–^ species, respectively, are indicating their coexistence on Zn(Pb).^[^ ^40^ ^]^ Another two weak bands near 1836 and 1740 cm^–1^ were observed between −0.8 and −1.2 *V*_RHE_ which refer to the bridge bonded CO ^[^ ^42^, ^44^ ^]^ and the adsorbed formyl *CHO species resulting in the first protonation of CO,^[^ ^40^ ^]^ respectively. Notably, an opposite trend of the 1660 cm^–1^ band can be observed in both samples, resulting in the *COOH and *OCOH intermediates enriched on the Zn surface depleted on Zn(Pb) surface. This indicates the desorption of CO‐driven intermediates on the Zn(Pb) surface as potential increased tuning the ECR selectivity. Furthermore, the *COOH band significantly decreased while the *OOCH band increased on Zn(Pb) surface with more negative potential. As a result, the *COOH/*OCOH and *OOCH intermediates have identified the pathway on CO‐selective Zn and HCOOH‐selective Zn(Pb) electrocatalysts, respectively, in agreement with the literature.^[^ ^45^, ^46^ ^]^ Furthermore, as the formate generation occurred by two‐oxygen bridge‐bonded formate intermediate (*OOCH); thus, it could be correlated to the cooperative Zn and Pb sites of Zn(Pb) surface forming asymmetric oxo‐bridge structure. Consequently, the Pb sites can modify the neighboring Zn's binding energy from being a C‐bonded site into an O‐bonded site boosting formate production rather than CO production.

### 2.4 Mechanistic Studies of the Electronic Structure due to the Alloying Effect

**Figure 4:**
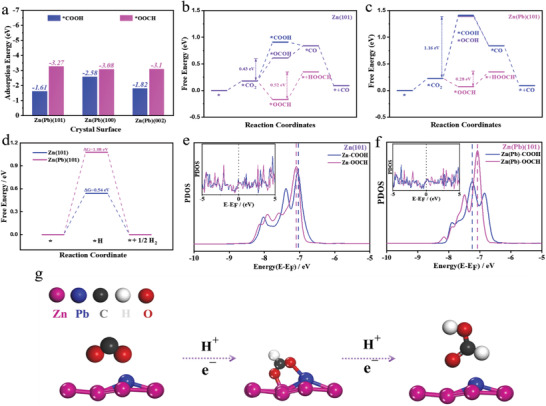
Theoretical calculations and reaction mechanism. Adsorption energy (eV) of different intermediates in Zn(Pb)(101), (100), (002) surface a). Free energy diagrams of ECR into CO/HCOOH on Zn(101) b) and Zn(Pb)(101) c) surfaces. Gibbs free energy calculation for HER pathway of Zn(101) and Zn(Pb)(101) d). Projected p‐orbital DOS of the Zn(101) e) and Zn(Pb)(101) f) sites with COOH* and *OOCH adsorbates (Inset figures: the PDOS near the Fermi level). Schematic illustration for CO_2_ reduction to formate on Zn(Pb) g).

To shed light on the catalytic mechanisms and confirm the selectivity control and activity enhancement, we performed DFT calculations and theoretical simulation of the intermediate's vibrations ( Table S4, Supporting Information). The adsorption energy (*E*_ads_) of *OOCH and *COOH were calculated as the most critical and observed intermediates on different interfaces, as shown in **Figure** **4**a. The *E*_ads_ of *OOCH and *COOH are the largest and lowest ones, respectively, on Zn(Pb)(101) surface, supporting the in situ FTIR results in which *OOCH is the predominant intermediate during the ECR reaction while *COOH species noticeably desorbed. According to the XRD results, Zn(Pb)(101) and its counter phase of Pb‐free Zn(101) were chosen under our DFT calculations. The configurations of intermediates, *OOCH (bridge), *COOH, and *OCOH (lay down *COOH), on Pb‐free Zn(101) and Zn(Pb)(101) were considered in our calculation and simulation ( Figure S3, Supporting Information). As shown in Figure 4b,c, the free energy diagram shows that Pb‐free Zn(101) surface prefer to be CO‐selective electrocatalysts due to the lowest free energy of the CO pathway barrier (Δ*G*_CO_ = 0.43 eV) by *OCOH intermediates. While on Zn(Pb)(101) surface, the free energy of the CO pathway barrier is extremely higher than that of the formate pathway barrier (Δ*G*_HCOOH_ = 0.28 eV). In other word, the free energy diagram demonstrates that Zn(Pb) surface prefers the adsorbed *OOCH species more than the *OCOH species due to the lowest free energy level of *OOCH species on Zn(Pb) surface. The adsorbed *OOCH and *OCOH species form the Zn–O–O–Pb structure which can be correlated to the synergetic effect between Zn and Pb as main active site and Pb–O–C–Zn structure over Pb as main reactive site, respectively. Therefore, the main chemical environment is the Zn–O–O–Pb structure resulting from the interaction modulation of the Zn sites to prefer O‐bonding mode instead of C‐bonding mode indicates that the synergetic effect between Zn and Pb is the main active site toward formate generation. Regarding the hydrogen evolution reaction (HER), Pb‐free Zn(101), and Zn(Pb)(101) sites show their advantages toward HER inhibition with weak adsorption of hydrogen molecule due to Zn sites, as seen in Figure 4d. The binding strength of selective CO and formate intermediates, i.e., (*COOH) and (*OOCH), could be concluded by comparing the projected density of states (PDOS) of the Pb‐free Zn(101) and Zn(Pb)(101) active sites with adsorbates, as shown in Figure 4e,f. The highest peak of active Pb‐free Zn(101) DOS of *COOH and *OOCH was closer to each other's with respect to the Fermi level, corresponding to lower filling of anti‐bonding states and hence more substantial adsorbate binding leading to energetically favored CO_2_‐to‐CO process.^[^ ^47^ ^]^ Regarding Zn(Pb)(101), the highest peak of *OOCH was closer than *COOH resulting energetically favored ECR process to produce formate. It rationalized the experimentally observed high FE_formate_ on Zn(Pb). As a result, Zn(Pb)(101) electronic structure could be correlated with ECR selectivity toward the formate generation pathway. The combination of Tafel analysis, detected intermediates, as well as DFT/DOS results, suggest that the proton‐assisted mechanism is the proposed pathway in the bimetallic Zn(Pb), as represented in Figure 4g, which involves coupling between adsorbed CO_2_ and either a surface hydrogen (*H) or a solvated proton (H^+^) species^[^ ^6^, ^48^, ^49^ ^]^ to form Zn–O–C–O–Pb structure. Our work demonstrates the correlation between the alloying aspect and the selectivity control/facilitation of the ECR products and sheds light on the effect of tuning the chemical environment on the electronic structure of the electrocatalysts.

## 3 Conclusion

In summary, an efficient ECR conversion toward highly selective formate product was obtained on Zn(Pb), showing an extreme Faradaic efficiency of ≈95%. Interestingly, an outstanding formate activity (47 mA cm^–2^) was recorded in a conventional H‐cell system. Having validated ECR performance, the flow cell electrolyzer was further designed, exhibiting an excellent activity stabilization on Zn(Pb) cathode. The origin of the efficient electroreduction of CO_2_–to–HCOOH stems from the effects of the asymmetric oxo‐bridged Zn–O–C–O–Pb bonds providing a favorable electronic structure for controlling CO selectivity and enhancing the ECR activity into formate compared to Zn‐ and Pb‐based electrocatalysts that were experimentally and theoretically confirmed. These findings will encourage the design of bimetallic catalysts that are able to efficiently tune the chemical environment achieving superior activity for CO_2_‐to‐formate reduction.

## 4 Experimental Section

### Chemicals

Zinc acetate dihydrate, lead acetate dihydrate, potassium hydroxide; KOH, Zn foil, and Cu foil were purchased from Sinopharm Chemical Reagent Co., Ltd. without treatments, potassium bicarbonate; KHCO_3_ (99.7% Sigma‐Aldrich), carbon papers (TGP‐H‐060), Nafion membrane (Nafion 211, Dupont), Nafion membrane solution (20%, Dupont), deionized water (purified by a Milli‐Q system) was used to prepare all solutions and to rinse samples and glassware.

### Characterizations

A high‐resolution SEM image of Zn(Pb) was obtained using a JEOL‐6700F. Morphological structure and elemental distribution were characterized by TEM(JEOL JEM‐2010) at the accelerating voltage of 200 kV. XRD patterns were recorded on a MiniFlex II diffractometer using a Cu K*α* (*λ* = 1.54 Å) radiation source at the scan rate of 0.5° min^–1^. The chemical valence and elemental surveying were analyzed by XPS (VG ESCALAB 250) with an Al K X‐ray source at 1487 eV. The chamber pressure was kept below 3 × 10^–10^ mbar during analysis and a specific correction was done by employing the C1s binding energy of 284.8 eV. ICP measurements were performed on an optical emission spectrometer (Horiba, Ultima 2) to detect sample atomic ratios. The in situ FTIR spectra in the wavenumber region 1200–2800 cm^–1^ were collected by a Fourier transform infrared spectrometer (Thermo scientific, Nicolet 6700 FTIR) with a ZnSe IR transparent window allowing the beam to pass through a thin electrolyte layer and to be reflected with an incidence angle of 65.

### Synthesis of Zn(Pb)

Three electrodes system was applied: Ag/AgCl (sat. KCl), Pt mesh (1 × 1 cm^2^), and carbon paper (0.5 × 0.5 cm^2^) were used as the reference, counter electrode, and working electrodes, respectively. The electrodeposition solution was composed of different atomic percentages of Pb and Zn by varying mole percentages of Pb^2+^ and Zn^2+^ in a total concentration equal to 0.2 m. The electrodeposition was set at cathode current density of 2.0 A cm^−2^ for the 40 s in a co‐electrodeposition solution containing 6 m KOH and 0.2 m of Zn(CH_3_COO)_2_ and Pb(CH_3_COO)_2_. A series of various Pb‐doped Zn were prepared using different concentrations of ion salts as listed in Table S1 in the Supporting Information.

### Electrochemical Measurements and Products Quantification

Electrochemical measurements were carried out in a three‐electrode system using an electrochemical station (CHI 660E). Ag/AgCl (saturated KCl) and Pt mesh (1 × 1 cm^2^) were used as reference and counter electrodes, respectively. The double‐layer capacitance of materials was evaluated in 0.1 m NaClO_4_ (99.5%, Aladdin) saturated with Ar. The LSV curve of ECR was obtained at a scan rate of 10 mV s^–1^. Electrochemical CO_2_ reduction reaction was carried out in an H‐type reactor filled with 0.1 m KHCO_3_ solution separated by a Nafion membrane. Furthermore, ECR in a two‐compartment built‐in house flow cell was performed wherein the anode (2 × 2 cm^2^ Ir/C; 300 µL of a mixture of 5 mg Ir/C, 950 µL isopropanol, and 50 µL Nafion (5%) on each side) and the cathode (0.5 × 0.25 cm^2^ Zn(Pb)) contained 0.5 m KHCO_3_, separated by anion exchange membrane (AEM). Prior to experiments, the cathodic electrolyte was pumped with CO_2_ (purity of 99.999%) for above 30 min, and CO_2_ was controlled to flow through the cathode compartment with the rate of 20 mL min^–1^ during experiments (except 10 mL min^–1^ for EIS). EIS was performed at opening potential with the frequency range from 0.01 Hz to 1 MHz and the voltage amplitude of 5 mV. The potential measured with reference electrode were converted to RHE scale with the equation

(3) $$E_{\mathrm{RHE}}=\;E_{\mathrm{Ag}/\mathrm{AgCl}}+0.197\;V+0.0591\;\times \mathrm{pH}$$

Gaseous products were sampled by the manual syringe and analyzed by the gas chromatography (GC, 9790II, FULI) equipped with a thermal conductivity detector and flame ionization detector. H_2_ and CO were detected using a TDX‐01 column. The carrier gas was Ar, and the quantification for each gas was determined by the external standard method. The FE calculation of CO was described as

(4) $$FE_{\mathrm{CO}}=\frac{\mathrm{CO}\;\mathrm{volume}\;÷\;22.4\mathrm{mol}\;L^{-1}\times \;2\;\times \;96485C\;\mathrm{mo}l^{-1}}{Q_{\mathrm{tot}}}$$

2 in the equation is the electron transfer number during every CO molecule generation. The FE calculation of H_2_ is similar to that of CO. Liquid products were analyzed by the ion chromatography (Sheng‐han CIC‐D100, Qingdao Shenghan). For the FE of HCOO^–^ calculating, the formula used is

(5) $$FE_{\mathrm{HCOO}-}=\frac{\frac{\mathrm{pp}m_{\mathrm{HCOO}}\times V_{\mathrm{Electrolyte}}}{M_{\mathrm{HCOO}-}}\times 2\times 96\;485\;C\;\mathrm{mo}l^{-1}}{Q_{tot}}$$

### Theoretical Calculations

Electronic Structures: The electronic structures of the understudied catalysts were computed by Vienna Ab‐initio Simulation Package (VASP). All calculations were performed with GGA‐PBE exchange‐correlation functional^[^ ^50^ ^]^ on periodically repeated slabs. All periodic slabs had a vacuum spacing of at least 10 Å. Computational models are as following: The Zn(101) supercell, made up by clave surface from Zn primitive cell and finally 3 × 4 × 3 atoms were applied in this model. The Zn(Pb)(101) model was simulated by replacing a top Zn atom with Pb. Additional phases (100) and (002) of Zn and Zn(Pb) were made in similar to (101) surface. All structures were fully relaxed to the ground state. The convergence of energy and forces were set to 1 × 10^−4^ and 0.05 eV, respectively. An energy cutoff is 400 eV, and the K‐point mesh is 3 × 3 × 1. The ground state structures of *COOH, *OCOH, *OOCH, *CO, and H* intermediates adsorbed on the Zn(101) and Zn(Pb)(101) were determined by searching the structure showed the lowest energy among all the possible configurations on the possible active sites. The ground state structures of the intermediate state on Zn(101)/Zn(Pb)(101) are shown in Figure S9 in the Supporting Information.

### XRD Simulation

An atom was replaced by Pb in the 3 × 3 × 2 atoms model of Zn supercell, and then the configuration and the lattice parameters were optimized, and the optimized model ( Figure S1, Supporting Information) was found. X‐powder diffraction simulation was carried on Reflex Tools in Material Studio 2019. In this section, because of the changes made in the lattice parameters, the k‐point mesh was modified to 5 × 5 × 7 and all structures were fully relaxed to the ground state. An energy cutoff was 400 eV and the convergence of energy and forces were set to 1 × 10^−4^ and 0.05 eV, respectively.

### Free Energy Calculation

Free energy calculation is based on the computational hydrogen electrode (CHE) model reported previously.^[^ ^51^, ^52^ ^]^ According to the CHE model, the free energy of a proton/electron pair (H^+^+e^−^) is equal to half of the gaseous hydrogen (1/2 H_2_) at an equilibrium potential for each process. Based on this model, the most comprehensive reaction pathways were considered toward the formation of CO and HCOOH products corresponding to two‐electron transfer for each CO_2_ molecule. The free energy of a species is calculated according to

(6) $$G=E_{\mathrm{DFT}}+E_{\mathrm{ZPE}}+\int \mathrm{CpdT}-\mathrm{TS}$$

where the *E*_DFT_, *E*_ZPE_, *C*p, *T*, *S* are the total energy, zero‐point energy, heat capacity, temperature, and entropy, respectively. The entropies of free molecular can be taken from the NIST data base,^[^ ^53^ ^]^ and the energy contribution from the configuration entropy in the adsorbed state was not included. A correction was made for CO (−0.51 eV) to the limitation of large deviations from the standard value in using GGA‐PBE functional. In addition, the solvation effects in the aqueous solution were considered: −0.11 eV was added to each O atom in intermediates in an approximate method. For example, the extra energy of −0.11 and −0.22 eV as a correction was added to *CO and *COOH/*OCOH/COOH intermediates.^[^ ^54^ ^]^ In addition, all corrections were carried on at 298 K and 1 atm. The elementary reactions with the change in the free energy in ECR considered are listed as follows

(7) $$*+{\mathrm{CO}}_{2(g)}+2\left(H^{+}+e^{-}\right)\to *+{\mathrm{CO}}_{\left(g\right)}$$

(8) $$*+{\mathrm{CO}}_{2(g)}+2\left(H^{+}+e^{-}\right)\to *+\mathrm{HCOO}H_{\left(l\right)}$$

1. CO pathways:

Pathway 1

(9) $$*+{\mathrm{CO}}_{2(g)}+e^{-}\to *\mathrm{CO}O^{-}$$

(10) $$*\mathrm{CO}O^{-}+H^{+}\to *\mathrm{COOH}$$

(11) $$*\mathrm{COOH}+H^{+}+e^{-}\to *\mathrm{CO}+H_{2}O_{\left(l\right)}$$

(12) $$*\mathrm{CO}\to *+CO_{\left(g\right)}$$

Pathway 2

(13) $$*+{\mathrm{CO}}_{2(g)}+e^{-}\to *\mathrm{CO}O^{-}$$

(14) $$*\mathrm{CO}O^{-}+H^{+}\to *\mathrm{OCOH}$$

(15) $$*\mathrm{OCOH}+H^{+}+e^{-}\to *\mathrm{CO}+H_{2}O_{\left(l\right)}$$

(16) $$*\mathrm{CO}\to *+CO_{\left(g\right)}$$

1. HCOOH pathway
(17) $$*+{\mathrm{CO}}_{2(g)}+e^{-}\to *\mathrm{CO}O^{-}$$
(18) $$*\mathrm{CO}O^{-}+H^{+}\to *\mathrm{OOCH}$$
(19) $$*\mathrm{OOCH}+H^{+}+e^{-}\to *+\mathrm{HCOO}H_{\left(l\right)}$$

The change in the free energy in HER considered are listed as follows

(20) $$*+\left(H^{+}+e^{-}\right)\to *H$$

(21) $$*H\to *+1/2\;H_{2}$$

### Adsorption Energy Calculation

Adsorption energy calculation according to

(22) $$E_{\mathrm{ads}}=E_{\left(\mathrm{ads}/\mathrm{metal}\right)}-E_{\left(\mathrm{metal}\right)}-E_{\left(\mathrm{ads}\right)}$$

where the *E*_ads_ is adsorption energy, *E*_(ads/metal)_, *E*_(metal)_, *E*_(ads)_ are the ground state energy of the total system (adsorbate+slab), metal, and absorbate.

### Vibrational Frequencies Calculation

The vibrational frequencies of adsorbates or molecules were calculated by taking harmonic approximation. In this study, IBRION = 5 was used to determine the Hessian matrix and the frequencies of absorbates, and the central differences (NFREE = 2) were carried on, which means all atoms were displaced in all three Cartesian directions and displaced by a small positive and negative displacement, resulting in a significant computational effort even for moderately sized high symmetry systems. In addition, slab was fixed when carrying on frequency calculation. Besides, the optimal range (900–2500 cm^–1^) was chosen according to the in situ IR's limitation (1200–2500 cm^–1^).

### Projected DOS Calculation

PDOS of Zn(101) and Zn(Pb)(101) surface were calculated by VASP on the previously optimized slab model. The further PDOS calculation on slab adsorbates (*COOH or *OOCH) was based on the previously optimized model with intermediate. Both the PDOS including all Zn atoms’ density of state in d orbital. K‐point mesh is 3 × 3 × 1 and all structures were fully relaxed to the ground state. An energy cutoff was 400 eV and the convergence of energy and forces were set to 1 × 10^−4^ and 0.05 eV, respectively.

## Conflict of Interest

The authors declare no conflict of interest.

## Supporting information

Supporting Information

## Data Availability

Research data are not shared.
