# Supplementary material for: Asymmetric Oxo‐Bridged ZnPb Bimetallic Electrocatalysis Boosting CO2‐to‐HCOOH Reduction
Source: Adv Sci (Weinh). 2021 Nov 10;9(4):2104138. doi: 10.1002/advs.202104138 (PMC8811806; doi:10.1002/advs.202104138)
Supplement: Supplementary file 1 — Supporting Information [file ADVS-9-2104138-s001.pdf]

WILEY-VCH

© 2021 Wiley-VCH GmbH

ADVANCED SCIENCE
Open Access

Supporting Information

for *Adv. Sci.,* DOI: 10.1002/advs.202104138

Asymmetric oxo-bridged ZnPb bimetallic electrocatalysis boosting CO2-to-HCOOH reduction

*Aya Gomaa Abdelkader Mohamed, Enbo Zhou, Zipeng Zeng, Jiafang Xie, Dunfeng Gao, Yaobing Wang **

Supporting Information

Asymmetric oxo-bridged ZnPb bimetallic electrocatalysis boosting CO_2-to-HCOOH reduction

*Aya Gomaa Abdelkader Mohamed, Enbo Zhou, Zipeng Zeng, Jiafang Xie, Dunfeng Gao, Yaobing Wang **

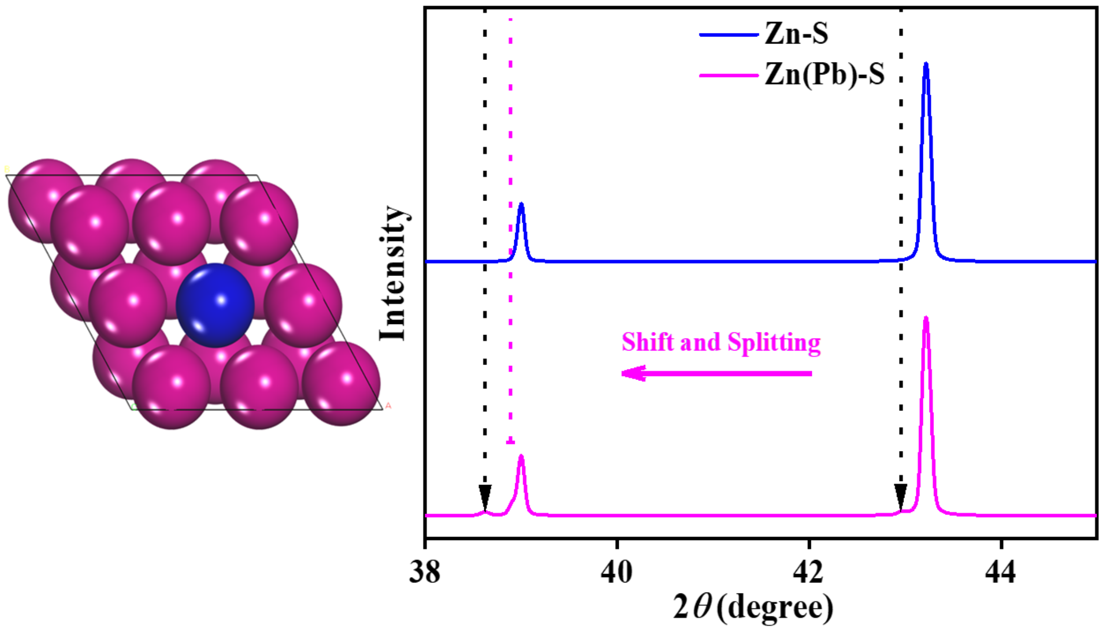

Figure S1 Optimized crystal of Zn for XRD simulation and the simulated XRD pattern.

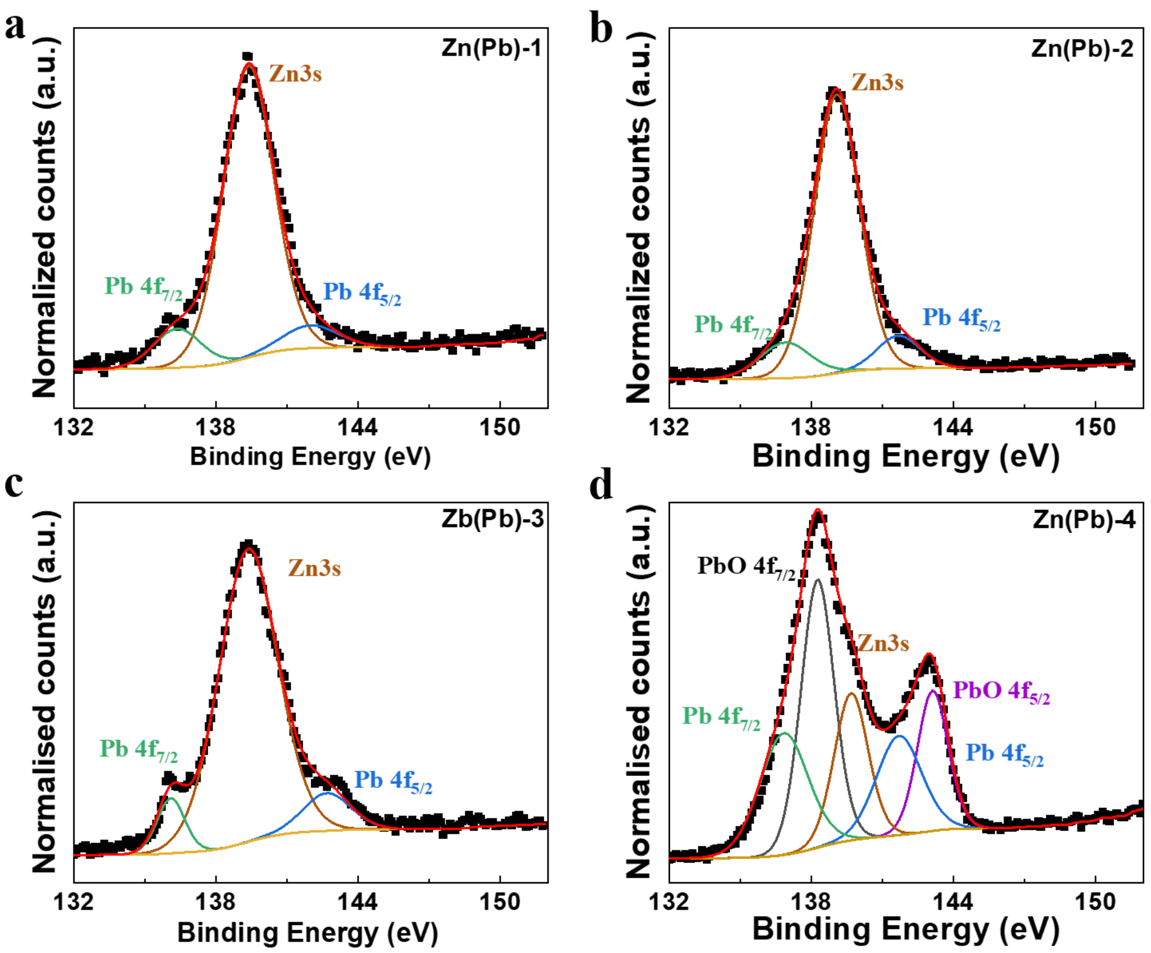

Figure S2 High resolution XPS of doped Zn samples.

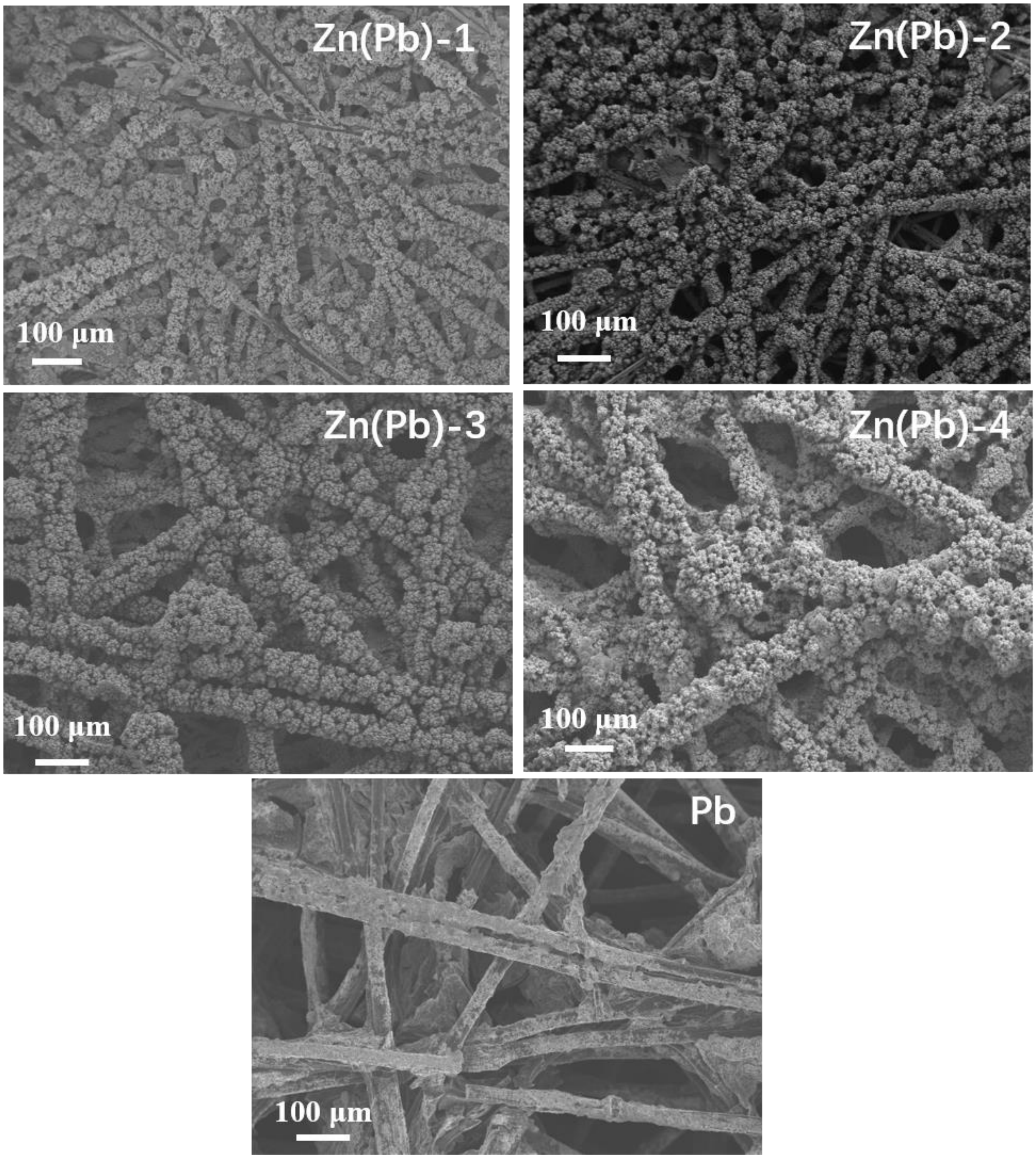

Figure S3 SEM images of the Pb-alloyed Zn electrodes.

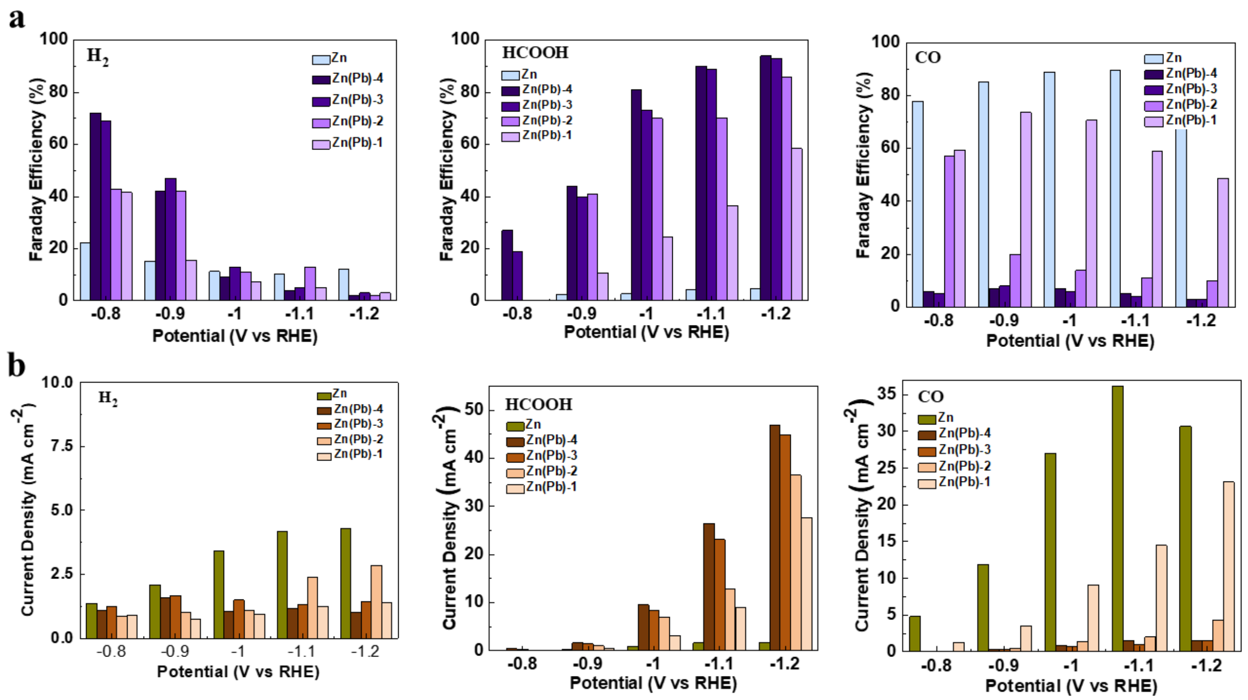

Figure S4 a) Faradaic efficiencies for H_2, HCOOH and CO formation on Zn(Pb) electrodes and their corresponding partial current densities b) at various potentials in comparison with undoped Zn electrode.

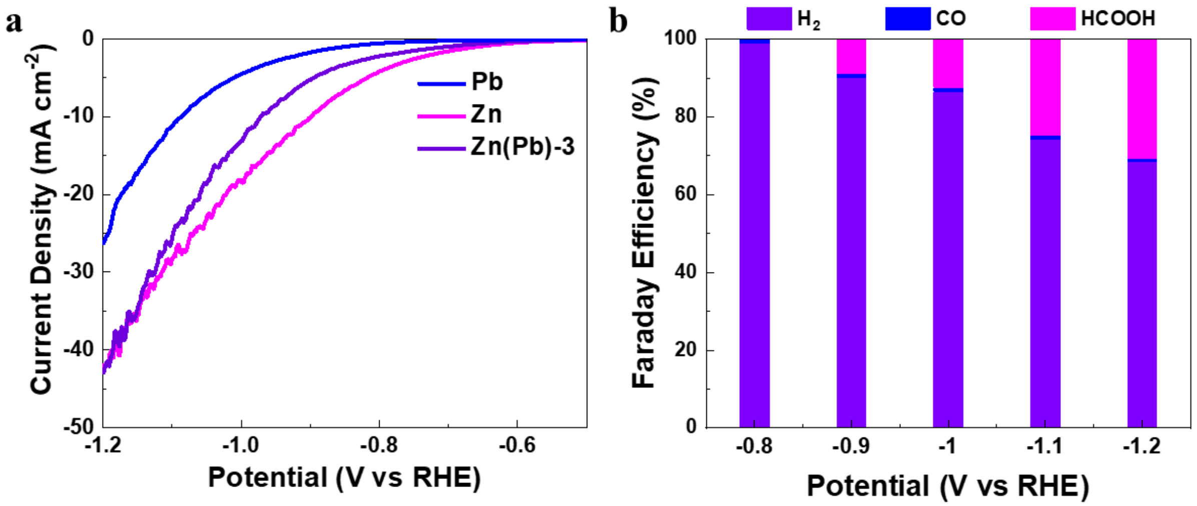

Figure S5 a) Current densities of as-prepared electrodes in CO_2-saturated 0.1 M $KHCO_3$ solution at various applied potentials. b) FE for $HCOO^-$, CO, H_2 products on Pb electrode.

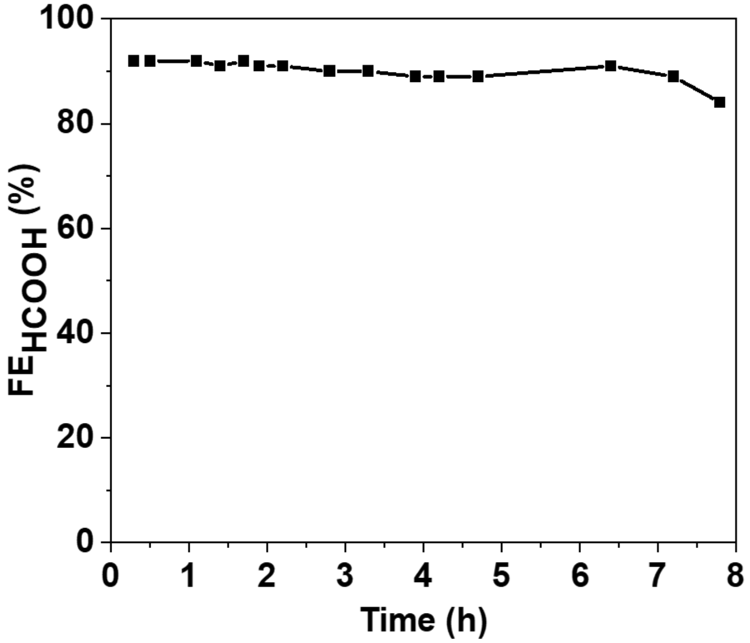

Figure S6 FE stability on Zn(Pb)-3 in H-cell.

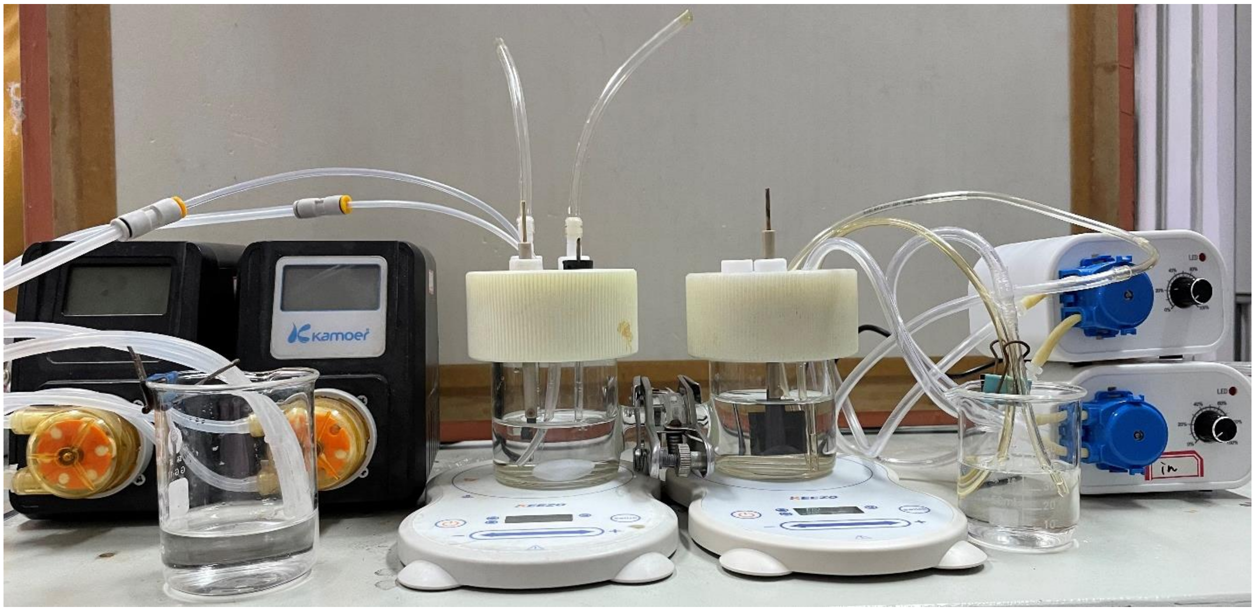

Figure S7 Flow cell setup used in the ECR electrocatalysis.

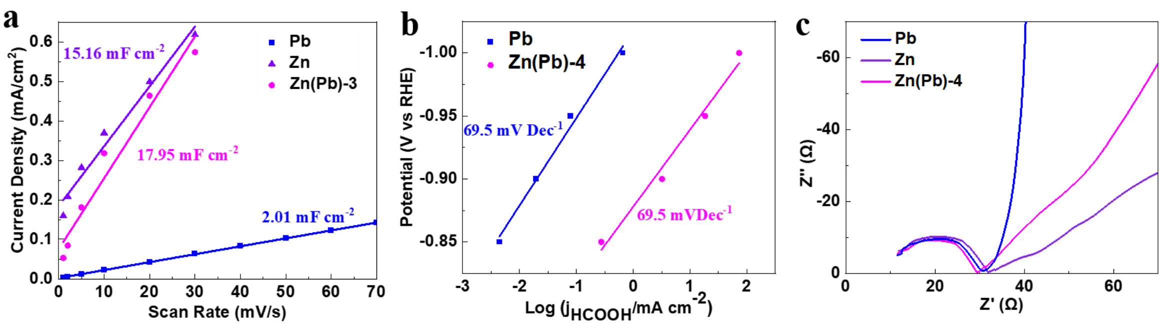

Figure S8 a) Charging current density differences plotted against scan rate, b) Tafel plot for producing HCOO-, and c) Nyquist plots for EIS analysis of as-prepared catalysts.

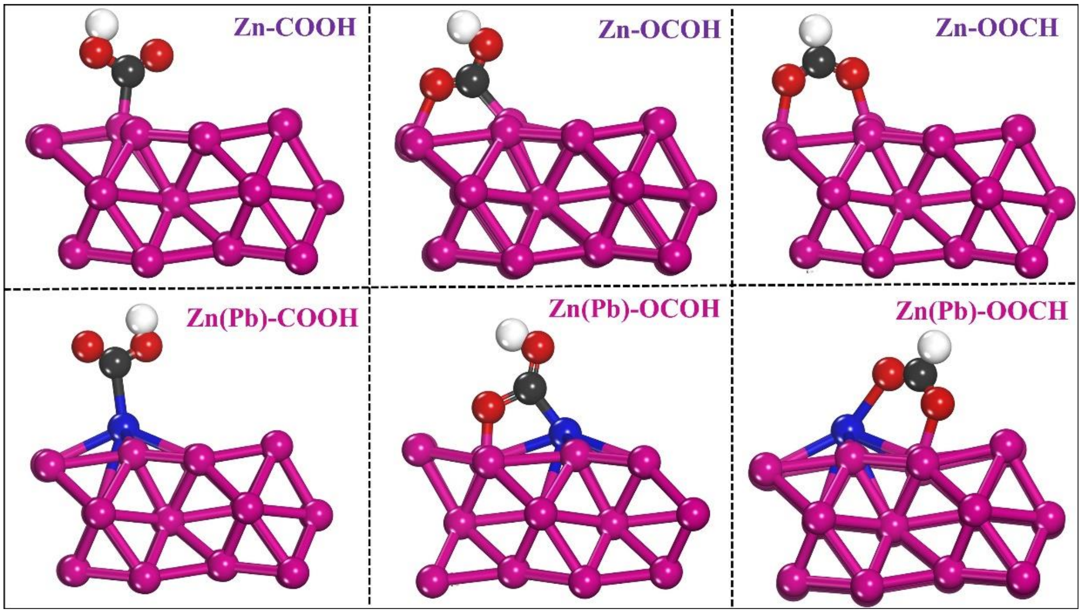

Figure S9 Configurations of *COOH/*OCOH/*OOCH in Zn(101) and Zn(Pb)(101) surface after optimized into ground state.

Table S1 Ratios of metals salts concentration with their corresponding labels.

**Labels of Zn(Pb)**	**Zn^{2+}: Pb^{2+} ratios**	**Pb content using ICP (%)**	**Pb content using EDS (%)**
Zn(Pb)-1	1999:1	-	-
Zn(Pb)-2	999:1	0.3	0.16
Zn(Pb)-3	99:1	0.7	0.92
Zn(Pb)-4	19:1	6.85	9.6

Table S2 Comparison of the activities Zn-based catalysts toward formate production in aqueous media using H-cell.

**Catalysts**	**Electrolyte**	**Electrolysis potential (V *vs*. RHE)**	**Partial current density (mA cm^{-2})**	**FE (%)**	**Reference**
RAD-Zn	0.5 M $NaHCO_3$	-1.9	12.8	87	[1]
CuZn-0.5	0.1 M $KHCO_3$	-1.1	4.5	60	[2]
$Zn_{0.95}In_{0.05}$	0.5 M $KHCO_3$	-1.2	22.5	95	[3]
ZnCu@Cu	0.5 M $NaHCO_3$	-1.25	22.5	48.6	[4]
$ZnBi_3$	0.5 M $NaHCO_3$	-0.8	3.8	94	[5]
O-Zn	0.1 M $KHCO_3$	-1.1	12.5	36.6	[6]
$ZnIn_2S_4$	0.1 M $KHCO_3$	-1.2	3	91	[7]
Zn(Pb)	0.1M $KHCO_3$	-1.2	47	95	This work

Table S3 Comparison of the activities Pb-based catalysts toward formate production in aqueous media.

**Catalysts**	**Electrolyte**	**Electrolysis potential (V *vs.* RHE)**	**Partial current density (mA cm^{-2})**	**FE (%)**	**Reference**
Pb	0.5 M $KHCO_3$	-1.17	3.96	72	[8]
Pb granules	0.5 M $KHCO_3$	-1.03	0.836	74	[9]
Pb plate	0.5 M NaOH	-0.86	2.5	65	[10]
Pb dendrites	1 M KHCO3	-0.99	7.5	97	[11]
Pulsed Pb	1 M KHCO3	-1.1	0.9	50	[12]
Nanolayered Pb	0.1 M KHCO3	-1.1	N/A	94	[13]
Oxide-derived Pb	0.5 M $NaHCO_3$	-0.8	0.6	98	[14]
Porous Pb	1 M $KHCO_3$	-0.99	8	97	[11]
0.78 Ml Pb/Cu	0.1M $KHCO_3$	-1.14	N/A	74	[15]
PbO_x@Cu_yO	0.5 M $NaHCO_3$	-0.9	20	60	[4]
Cu–Pb	0.5M $KHCO_3$	-0.93	2	20	[16]
Amine modified Pb	1 M $KHCO_3$	-1.29	24	80	[17]
$Sn_{56.3}Pb_{43.7}$	0.5M $KHCO_3$	-1.4	45.7	80	[18]
Oxide-derived Sn-Pb-Sb	0.1 M $KHCO_3$	-1.4	8.3	91	[19]
Sulfide-derived Pb	0.1M $KHCO_3$	-1.08	12	88	[20]
TA-Pb	0.5 M $NaHCO_3$	-0.92	0.4	96	[21]
Zn(Pb)	0.1M $KHCO_3$	-1.2	47	95	This work

Table S4 Calculated vibrational frequencies (900~2500 cm^{-1}) of adsorbates and CO_2.

Crystal Surface	Intermediate	Frequency / cm^{-1}		
Zn(101)	*COOH	1493.03	1267.50	1092.79
	*OCOH	1655.21	1230.90	1005.50
	*OOCH	1536.81	1353.89	1331.25
Zn(100)	*COOH	1674.88	1229.88	1003.44
	*OCOH	1504.42	1254.85	1094.52
	*OOCH	1536.60	1343.95	1328.97
Zn(102)	*COOH	1667.47	1224.07	992.65
	*OCOH	1511.64	1265.85	1105.49
	*OOCH	1538.77	1340.21	1330.84
Zn(Pb)(101)	*COOH	1517.53	1268.21	1093.31
	*OCOH	1698.63	1228.15	979.94
	*OOCH	1537.71	1339.37	1301.07
Zn(Pb)(100)	*COOH	1710.91	1231.33	991.71
	*OOCH	1538.78	1334.56	1325.18
Zn(Pb)(002)	*COOH	1706.18	1240.26	986.49
	*OOCH	1532.95	1338.55	1317.36
CO_2		2365.55		1324.55

References

[1] T. T. Zhang, H. X. Zhong, Y. L. Qiu, X. F. Li, H. M. Zhang, *J. Mater. Chem. A* **2016**, *4*, 16670-16676.

[2] S. Ajmal, Y. Yang, K. J. Li, M. A. Tahir, Y. Y. Liu, T. Wang, A. U. R. Bacha, Y. Q. Feng, Y. Deng, L. W. Zhang, *J. Phys. Chem. C* **2019**, *123*, 11555-11563.

[3] I. S. Kwon, T. T. Debela, I. H. Kwak, H. W. Seo, K. Park, D. Kim, S. J. Yoo, J. G. Kim, J. Park, H. S. Kang, *J. Mater. Chem. A* **2019**, *7*, 22879-22883.

[4] V. S. S. Mosali, X. L. Zhang, Y. Zhang, T. Gengenbach, S. X. Guo, G. Puxty, M. D. Horne, A. M. Bond, J. Zhang, *ACS Sustainable Chem. Eng.* **2019**, *7*, 19453-19462.

[5] T. T. Zhang, Y. L. Qiu, P. F. Yao, X. F. Li, H. M. Zhang, *ACS Sustainable Chem. Eng.* **2019**, *7*, 15190-15196.

[6] T. L. Chen, H. C. Chen, Y. P. Huang, S. C. Lin, C. H. Hou, H. Y. Tan, C. W. Tung, T. S. Chan, J. J. Shyue, H. M. Chen, *Nanoscale* **2020**, *12*, 18013-18021.

[7] Z. T. Wang, R. J. Qi, D. Y. Liu, X. D. Zhao, L. Huang, S. H. Chen, Z. Q. Chen, M. T. Li, B. You, Y. J. Pang, B. Y. Xia, *ChemSusChem* **2021**, *14*, 852-859.

[8] Y. Hori, K. Kikuchi, S. Suzuki, *Chem. Lett.* **1985**, *14*, 1695-1698.

[9] F. Koleli, T. Atilan, N. Palamut, A. M. Gizir, R. Aydin, C. H. Hamann, *J. Appl. Electrochem.* **2003**, *33*, 447-450.

[10] B. Innocent, D. Liaigre, D. Pasquier, F. Ropital, J. M. Leger, K. B. Kokoh, *J. Appl. Electrochem.* **2009**, *39*, 227-232.

[11] M. Fan, S. Garbarino, G. A. Botton, A. C. Tavares, D. Guay, *J. Mater. Chem. A* **2017**, *5*, 20747-20756.

[12] M. J. W. Blom, V. Smulders, W. P. M. van Swaaij, S. R. A. Kersten, G. Mul, *Appl. Catal. B-Environ.* **2020**, *268*, 7.

[13] Y. Kwon, J. Lee, *Electrocatalysis* **2010**, *1*, 108-115.

[14] C. H. Lee, M. W. Kanan, *ACS Catal.* **2015**, *5*, 465-469.

[15] C. Kim, T. Möller, J. Schmidt, A. Thomas, P. Strasser, *ACS Catal.* **2019**, *9*, 1482-1488.

[16] Y. Wang, H. Hu, Y. Sun, Y. Tang, L. Dai, Q. Hu, A. Fisher, X. J. Yang, *Adv. Mater. Interfaces* **2019**, *6*.

[17] N. Zouaoui, B. D. Ossonon, M. Fan, D. Mayilukila, S. Garbarino, G. de Silveira, G. i. A. Botton, D. Guay, A. C. Tavares, *J. Mater. Chem. A* **2019**, *7*, 11272-11281.

[18] S. Y. Choi, S. K. Jeong, H. J. Kim, I. H. Baek, K. T. Park, *ACS Sustainable Chem. Eng.* **2016**, *4*, 1311-1318.

[19] S. Rasul, A. Pugnant, H. Xiang, J. M. Fontmorin, E. H. Yu, *J. CO_2 Util.* **2019**, *32*, 1-10.

[20] J. E. Pander, J. W. J. Lum, B. S. Yeo, *J. Mater. Chem. A* **2019**, *7*, 4093-4101.

[21] Y. M. Shi, Y. Ji, J. Long, Y. Liang, Y. Liu, Y. F. Yu, J. P. Xiao, B. Zhang, *Nat. Commun.* **2020**, *11*, 10.
